# Effect of Messaging on Support for Breast Cancer Screening Cessation Among Older US Women: A Randomized Clinical Trial

**DOI:** 10.1001/jamanetworkopen.2024.28700

**Published:** 2024-08-19

**Authors:** Nancy L. Schoenborn, Sarah E. Gollust, Rebekah H. Nagler, Craig E. Pollack, Cynthia M. Boyd, Qian-Li Xue, Mara A. Schonberg

**Affiliations:** 1 Department of Medicine, Division of Geriatric Medicine and Gerontology, Johns Hopkins University School of Medicine, Baltimore, Maryland; 2 Division of Health Policy and Management, University of Minnesota School of Public Health, Minneapolis; 3 University of Minnesota Hubbard School of Journalism and Mass Communication, Minneapolis; 4 Department of Health Policy and Management, Johns Hopkins University Bloomberg School of Public Health, Baltimore, Maryland; 5 Johns Hopkins Center on Aging and Health, Baltimore, Maryland; 6 Division of General Medicine and Primary Care, Beth Israel Deaconess Medical Center, Harvard Medical School, Brookline, Massachusetts

## Abstract

**Importance:**

Many older women are screened for breast cancer beyond guideline-recommended thresholds. Messaging holds promise to reduce overscreening.

**Objective:**

To investigate the effect of a message on older women’s support for and intentions of stopping breast cancer screening.

**Design, Setting, and Participants:**

A 2-wave randomized clinical online survey trial using a nationally representative online panel was performed from May 12 to June 19, 2023. Women 65 years or older without breast cancer were eligible to participate.

**Intervention:**

A pilot-tested breast cancer screening cessation message delivered to a hypothetical older woman with serious illnesses and functional impairment. The message was described as from 1 of 3 sources (clinician, news story, or family member). Participants were randomized into 4 groups: no message (group 1 [control]), a single message from a clinician at wave 1 and no message at wave 2 (group 2), a message from a news story (wave 1) and a clinician (wave 2) (group 3), and a message from a family member (wave 1) and a clinician (wave 2) (group 4).

**Main Outcomes and Measures:**

Support for stopping screening in the hypothetical older woman (primary) and screening intentions for oneself (secondary) were assessed on 7-point scales, with higher values indicating stronger support for and intentions to stop screening. Means were compared using analysis of variance. The message effect on screening intentions among participants 75 years or older and those with life expectancy of less than 10 years were also explored.

**Results:**

A total of 3051 women participated in wave 1 of the trial. The mean (SD) age was 72.8 (5.9) years; 272 (8.9%) were non-Hispanic Black and 2506 (82.1%) were non-Hispanic White. Of these women, 2796 (91.6%) completed wave 2. Group 2 had significantly higher support for screening cessation in the hypothetical patient at wave 2 (mean score, 3.14 [95% CI, 2.99-3.29]) compared with group 1 (mean score, 2.68 [95% CI, 2.54-2.82]; *P* < .001). The effect was even stronger in group 3 (mean score, 4.23 [95% CI, 4.09-4.38]) and group 4 (mean score, 4.12 [95% CI, 3.97-4.27]) compared with both groups 1 and 2 (all *P* < .001). Message effects on self-screening intentions followed a similar pattern, with larger effects among participants 75 years or older or with limited life expectancy.

**Conclusions and Relevance:**

In this randomized clinical trial, a breast cancer screening cessation message significantly increased older women’s support for and intentions of screening cessation. The strongest effects were observed when the message was delivered over time from multiple sources. Future work needs to engage potential message sources to examine the feasibility and acceptability of multilevel messaging strategies and their effect on screening behavior.

**Trial Registration:**

ClinicalTrials.gov Identifier: NCT05821023

## Key Points

**Question:**

Does a message describing rationales for stopping breast cancer screening affect older women’s support for appropriate screening cessation?

**Findings:**

This 2-wave randomized clinical trial of older women (3051 in wave 1, 2796 in wave 2) used an online survey to assess support for stopping screening in a hypothetical patient and self-screening intentions. A message about stopping screening from a clinician significantly increased support for appropriate screening cessation, with stronger effects (ie, an increase from 18% to 47%) when the message was delivered over time from multiple sources.

**Meaning:**

These findings suggest that messaging is a viable strategy for reducing overscreening for breast cancer among older women.

## Teaser

This randomized clinical trial evaluates whether receipt of consistent messages about cessation of breast cancer screening from nonclinician sources may amplify the message effect among older women with limited life expectancy compared with receiving the message from a clinician alone.

## Introduction

Although mammography screening can reduce breast cancer–related mortality and morbidity, it can also cause harms such as false-positive findings and overdiagnosis.^1,2,3,4^ The benefits of mammography screening lag by about 10 years, whereas the harms and burdens of screening occur in the short term.^1,2,3,4,5^ In older women with multiple chronic conditions, functional impairment, and limited life expectancy, the harms of screening likely outweigh the benefits.^1,2,3^ Guidelines recommend screening up to 75 years of age or as long as life expectancy is 10 years or greater.^6,7,8^ However, many older women continue to receive screening beyond these thresholds.^9,10,11,12^ National data found that 50.6% of women 75 years or older and 42.8% of women with a life expectancy of less than 10 years received breast cancer screening, suggesting overscreening.^10^

One important contributor to overscreening is that patients have received proscreening messages for many years from the media, the broader social environment, and health care professionals.^13,14,15,16^ In contrast, there has been little messaging about the harms of overscreening or that stopping screening may be appropriate for some women. Messaging strategies have been used successfully to reduce other unwanted health behaviors such as smoking but are an understudied approach to reduce overscreening.^17,18^ In prior work, we developed and evaluated messages on breast cancer screening cessation for older women.^19^ We combined the top-rated message elements from this prior work into a single message in the present study to examine whether message exposure would increase support for and intentions of stopping screening.

When considering effective communication strategies, message source also matters. Older adults consider clinicians the most trusted information source about cancer screening, but family members, friends, and the media are also important.^13,14,15,16,19,20,21,22,23,24^ Therefore, we also sought to experimentally evaluate whether receiving consistent messages about stopping screening from nonclinician sources may amplify the message effect compared with receiving a message from a clinician alone.

## Methods

### Study Overview

We conducted a 2-wave, randomized clinical online survey experiment with women 65 years and older. We included women aged 65 to 75 years because women in this age group with multiple chronic conditions and functional impairments may have a life expectancy of less than 10 years.^25^ Furthermore, the message may prime women to consider stopping screening in the future and/or influence others in their social network.

Participants were randomized into groups that varied in the number of messages they received over 2 points (ie, 0, 1, or 2), the source of the messages (ie, clinician only, a news story plus a clinician, or a family member plus a clinician), and the message content (ie, promoting screening cessation vs screening continuation). In this study, we only report on the effects of the screening cessation message (the effects of conflicting messages wherein participants were exposed to both screening cessation and continuation message contents will be reported elsewhere). This study followed the Consolidated Standards of Reporting Trials (CONSORT) reporting guideline and the Checklist for Reporting Results of Internet E-Surveys (CHERRIES). This study was approved by the Institutional Review Board of Johns Hopkins University School of Medicine, and all participants provided consent via completion of the survey. The trial protocol is provided in Supplement 1.

Recruitment used a nationally representative, probability-based online panel (KnowledgePanel).^26^ Panel members are recruited by random digit dialing (until 2009) and address-based sampling (since 2009). Panel members were invited to participate via email if they were women 65 years or older, were English-speaking, and had no history of breast cancer. Each received a unique survey link that could only be used once. The study ended once the target sample size was reached. Panel members received incentives from KnowledgePanel using a point system. The first wave of the survey was administered between May 12 and 30, 2023, and the second wave was administered between May 30 and June 19, 2023.

### Survey Instrument

The survey instrument was developed by the study team and cognitively tested with 6 older women prior to study initiation; we iteratively revised the instrument wordings based on their feedback. The survey described a hypothetical 75-year-old woman with serious health problems and functional limitations but who was not imminently dying (eAppendix in Supplement 2). We used this hypothetical scenario to provide a standardized and relevant context of an older woman with a life expectancy of less than 10 years for whom it would be appropriate to stop screening.^7,8^ We mentioned that the hypothetical patient had no history of breast cancer and had regular mammograms and that her most recent mammogram results were normal.

We then randomized exposure to a message that included rationales for stopping screening. Based on our prior work developing and evaluating messages on stopping screening,^19^ the message in this study mentioned guideline recommendations, an anecdote about women who experienced false-positive results, and evidence on overdiagnosis. We described this message as from one of 3 sources: (1) the hypothetical patient’s primary care physician, who shared information during a clinic visit; (2) a news story that the hypothetical patient read in *USA Today*; or (3) the hypothetical patient’s close family member who shared information during a social visit. We made minor modifications based on message source; for example, the message from the clinician said, “I have several older patients,” whereas the message from the family member said, “I know several older women.” Complete survey details are provided in the eAppendix in Supplement 2.

### Experimental Design and Outcomes

**Figure 1.**
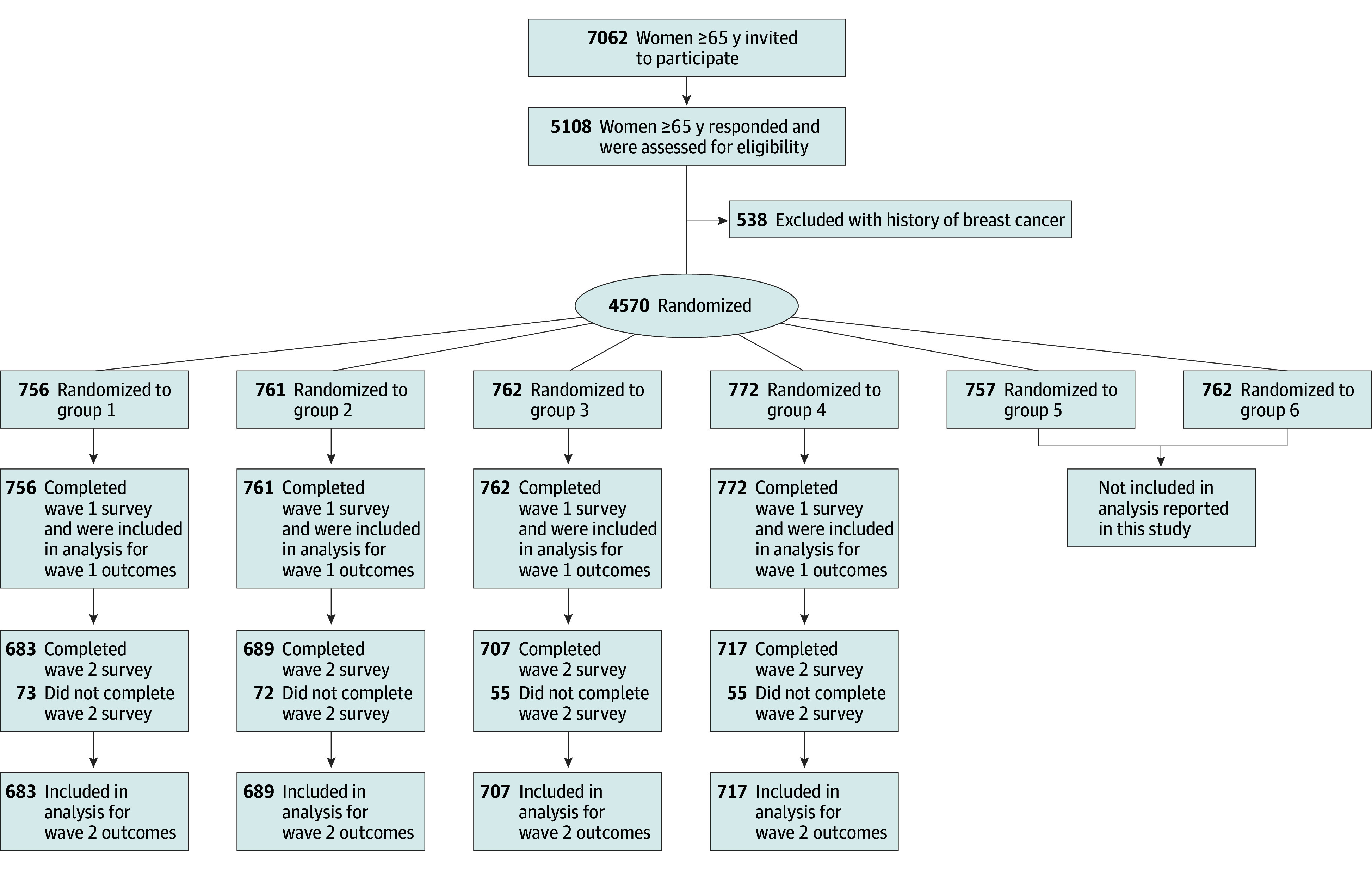
Participant Flow and Study Design Group 1 received no messages; group 2, a message from a clinician only in wave 1; group 3, messages from a news story in wave 1 and a clinician in wave 2; and group 4, messages from a family member in wave 1 and a clinician in wave 2.

All participants viewed the patient vignette and then were randomized into 4 groups (Figure 1). To ensure matched characteristics across groups, participants were sorted by self-reported race and ethnicity, geographic region, and educational level (using information collected about KnowledgePanel members previously), and then a random number generated by survey programming allocated participants sequentially to each experimental group. The research team was blinded to the allocation until data collection was complete.

Group 1 (the control group) received no message at either wave. Group 2 received the message on stopping screening from the clinician at wave 1 and no message at wave 2. Group 3 received the message on stopping screening from a news story at wave 1 and the same message from the clinician at wave 2. Group 4 received the message on stopping screening from the family member at wave 1 and the same message from the clinician at wave 2. By presenting the nonclinician message first, we aimed to mimic a scenario wherein patients may be exposed to information from their social networks and/or the media before a clinic visit.

Our primary outcome was support for screening cessation: whether participants believed that older women like the hypothetical patient (ie, someone for whom it would be appropriate to stop screening) should get a mammogram in the next 2 years, measured on a 7-point scale (1 indicates definitely should get a mammogram; 7, definitely should not get a mammogram). Our secondary outcome consisted of participants’ screening cessation intentions for themselves in the next 2 years, also measured on a 7-point scale (1 indicates very likely to get a mammogram; 7, very unlikely to get a mammogram). Both outcomes were assessed at both waves in all groups.

We also assessed other consequences of message exposure, all measured on a 5-point Likert scale at wave 1. We assessed the extent that the message might cause a general negative perception of mammograms. This was a consequence we aimed to avoid since our goal was to inform women of the potential harms of overscreening rather than dissuade all women from mammograms. We asked whether the message would “discourage” one from getting mammograms, make getting mammograms “unpleasant,” or make one “concerned about the health effects” of getting mammograms (1 indicates strongly disagree; 5, strongly agree).^27^ We also assessed 2 negative emotional responses: whether the message made one feel “annoyed” or “worried” (1 indicates not at all; 5, extremely). These outcomes were only assessed in groups 2 to 4.

The survey instrument also assessed prior breast cancer screening, Gail-model breast cancer risk factors (eg, family history, number of breast biopsies),^28^ cancer worry, health literacy,^29^ and health or functional status (used to estimate 10-year life expectancy).^25^ KnowledgePanel provided participants’ age, sex, race and ethnicity, educational level, and geographic region. Only deidentified data were provided. For select items, a repeat prompt was used if the item was skipped.

### Statistical Analysis

The analysis focused on (1) whether exposure to any message about stopping screening would increase support for stopping screening (ie, comparing groups 2-4 vs 1); and (2) whether exposure to consistent messages from 2 sources was more effective than exposure to a single message from the clinician (ie, comparing groups 3 and 4 vs 2). We focused on results at wave 2 to examine the cumulative effects of multiple message exposures. Informed by preliminary data, we estimated 400 participants per group at wave 2 would allow 80% power to detect a 0.30-point difference on the 7-point scale for the primary outcome at an α level of .05. Assuming 20% attrition between waves, we aimed to recruit 500 women per group. Due to high response rate in a shorter-than-expected time, actual recruitment exceeded planned sample size.

Regarding support for stopping screening for the hypothetical patient (primary outcome) and screening intention for oneself (secondary outcome), we compared the mean scores across groups at wave 2 using analysis of variance with adjusting for multiple comparisons using the Tukey test. For ease of interpretation, we also dichotomized these 2 outcomes where scores 5 to 7 were categorized as supports screening cessation and scores 1 to 4 as does not support screening cessation. In exploratory analysis, we examined screening intention for oneself at wave 2 among participants who met guideline thresholds for stopping screening—namely, participants 75 years or older and participants with predicted life expectancy of less than 10 years.^7,8,9^ We also explored screening intention for oneself at wave 2 among participants with higher breast cancer risk, defined as 5-year risk of at least 3%, which is the threshold used in guidelines for considering cancer prevention medications.^30,31^ For the other consequence outcomes, analyses focused on summarizing mean scores across groups. All analyses were performed using Stata, version 17 (StataCorp LLC). Two-sided *P* ≤ .05 indicated statistical significance.

## Results

This study included 3051 women in wave 1, of whom 1032 (33.8%) were 75 years or older and 2019 were aged 65 to 74 years (66.2%) (mean [SD] age, 72.8 [5.9] years) (Table 1). In terms of race and ethnicity, 148 women (4.9%) were Hispanic, 272 (8.9%) were non-Hispanic Black, 2506 (82.1%) were non-Hispanic White, and 125 (4.1%) were of other race or ethnicity (including non-Hispanic multiracial and non-Hispanic other). Overall, 7062 women had been invited to participate in the larger project, and 5108 (72.3%) responded at wave 1 and were assessed for eligibility. Compared with responders, nonresponders were slightly younger and less likely to be White (eTable 1 in Supplement 2). Of the responders, 538 women with breast cancer history were excluded; among the remaining 4570 participants, 3051 women were randomized to the groups relevant to the present study (ie, the control group 1 and experimental groups 2-4 that received screening cessation messages) and are included in the analysis below. Of these 3051 women, 2796 (91.6%) completed wave 2. Compared with noncompleters, wave 2 completers were less likely to be worried about breast cancer but were not otherwise different (eTable 2 in Supplement 2).

**Table 1.** Participant Characteristics

Characteristic	Participant group^a^				
	All (N = 3051)	Group 1 (n = 756)	Group 2 (n = 761)	Group 3 (n = 762)	Group 4 (n = 772)
Age, y					
65 to <75	2019 (66.2)	503 (66.5)	502 (66.0)	507 (66.5)	507 (65.7)
≥75	1032 (33.8)	253 (33.5)	259 (34.0)	255 (33.5)	265 (34.3)
Life expectancy, y^b^					
≥10	2437 (81.5)	593 (79.9)	617 (82.5)	619 (83.3)	608 (80.1)
<10	555 (18.5)	149 (20.1)	131 (17.5)	124 (16.7)	151 (19.9)
Race and ethnicity					
Hispanic	148 (4.9)	35 (4.6)	33 (4.3)	40 (5.2)	40 (5.2)
Non-Hispanic Black	272 (8.9)	71 (9.4)	73 (9.6)	63 (8.3)	65 (8.4)
Non-Hispanic White	2506 (82.1)	617 (81.6)	621 (81.6)	630 (82.7)	638 (82.6)
Other^c^	125 (4.1)	33 (4.4)	34 (4.5)	29 (3.8)	29 (3.8)
Geographic region					
Northeast	525 (17.2)	128 (16.9)	141 (18.5)	124 (16.3)	132 (17.1)
Midwest	723 (23.7)	179 (23.7)	185 (24.3)	180 (23.6)	180 (23.3)
South	1086 (35.6)	271 (35.8)	263 (34.6)	273 (35.8)	279 (36.1)
West	717 (23.5)	178 (23.5)	173 (22.7)	185 (24.3)	181 (23.4)
Ever had mammogram	2958 (97.0)	733 (97.0)	739 (97.1)	736 (96.6)	750 (97.2)
Self-reported mammogram within last 2 y^d^	2435 (79.9)	601 (79.7)	607 (79.8)	608 (80.0)	619 (80.2)
Cancer worry^e^					
Somewhat, a little, or not at all worried	2772 (91.1)	670 (88.7)	700 (92.1)	701 (92.4)	701 (91.0)
Moderately or extremely worried	272 (8.9)	85 (11.3)	60 (7.9)	58 (7.6)	69 (9.0)
Positive family history of breast cancer^f^	706 (23.8)	175 (24.1)	179 (24.1)	160 (21.5)	192 (25.5)
Educational level					
High school or less	860 (28.2)	211 (27.9)	212 (27.9)	212 (27.8)	225 (29.1)
Some college or more	2191 (71.8)	545 (72.1)	549 (72.1)	550 (72.2)	547 (70.9)
Low health literacy^g^	239 (7.9)	64 (8.5)	52 (6.9)	55 (7.3)	68 (8.8)
5-y Probability of breast cancer, mean (SD)^h^	2.4 (1.3)	2.4 (1.3)	2.4 (1.3)	2.3 (1.4)	2.4 (1.3)

^a^ Group 1 received no messages; group 2, a message from a clinician only in wave 1; group 3, messages from a news story in wave 1 and a clinician in wave 2; and group 4, messages from a family member in wave 1 and a clinician in wave 2. Unless otherwise indicated, data are expressed as No. (%) of participants. Some totals do not sum to 3051 due to incomplete or missing data.

^b^ Includes 2992 participants. Life expectancy was estimated using the Schonberg mortality index.^25^ Scores for participants ranged from 0 to 19. Scores of 10 or greater are associated with a greater than 50% chance of 10-year mortality. Thus, women who score 10 or greater are estimated to have a life expectancy of less than 10 years.

^c^ Includes non-Hispanic multiracial and non-Hispanic other race.

^d^ Includes 3047 participants.

^e^ Includes 3044 participants.

^f^ Includes 2966 participants.

^g^ Includes 3038 participants. Health literacy was assessed in a single validated question: “How confident are you filling out medical forms?”^29^ Responses of not at all, a little bit, and somewhat confident were categorized as low health literacy; responses of quite a bit and extremely confident were categorized as normal health literacy.

^h^ Includes 2948 participants. Based on Gail Breast Cancer Risk Assessment Tool.^26^

### Support for Screening Cessation for the Hypothetical Patient

At wave 1, the control group that received no message (group 1) had the lowest support for stopping screening for the hypothetical patient (Table 2), with a mean score of 2.66 (95% CI, 2.52-2.81) on the 7-point scale (with higher scores indicating stronger support for screening cessation). Receiving a screening cessation message at wave 1 from any source was associated with significantly higher support for screening cessation, with comparable effects when delivered by a clinician (mean score, 3.52 [95% CI, 3.37-3.67]) or a news story (mean score, 3.58 [95% CI, 3.44-3.72]) and a weaker effect when delivered by a family member (mean, 2.94 [95% CI, 2.81-3.08]) (*P* < .05 for all compared with group 1).

**Table 2.** Mean Outcome Scores^a^

	Participant group			
	Group 1	Group 2	Group 3	Group 4
**Support for stopping screening for hypothetical patient**				
Wave 1				
No. of participants	756	761	762	772
No. missing	4	3	2	4
Score (95% CI)	2.66 (2.52-2.81)	3.52 (3.37-3.67)	3.58 (3.44-3.72)	2.94 (2.81-3.08)
Wave 2				
No. of participants	683	689	707	717
No. missing	4	6	3	6
Score (95% CI)	2.68 (2.54-2.82)	3.14 (2.99-3.29)^b^	4.23 (4.09-4.38)^b^^,^^c^	4.12 (3.97-4.27)^b^^,^^c^
**Stopping screening intention for oneself**				
Wave 1				
No. of participants	756	761	762	772
No. missing	4	1	0	5
Score (95% CI)	2.48 (2.31-2.65)	2.81 (2.63-2.98)	2.82 (2.65-3.00)	2.52 (2.36-3.69)
Wave 2				
No. of participants	683	689	707	717
No. missing	3	3	4	2
Score (95% CI)	2.38 (2.21-2.55)	2.59 (2.42-2.77)	2.95 (2.77-3.13)^b^^,^^c^	2.98 (2.80-3.17)^b^^,^^c^
**Stopping screening intention for oneself at age ≥75 y**				
Wave 1				
No. of participants	253	259	255	265
No. missing	2	0	0	1
Score (95% CI)	2.96 (2.64-3.29)	3.41 (3.08-3.73)	3.61 (3.29-3.94)	3.22 (2.90-3.54)
Wave 2				
No. of participants	222	232	241	251
No. missing	1	2	2	1
Score (95% CI)	2.82 (2.49-3.16)	3.32 (2.98-3.66)	3.83 (3.50-4.16)^b^^,^^c^	3.80 (3.46-4.13)^b^^,^^c^
**Stopping screening intention for oneself with life expectancy <10 y**				
Wave 1				
No. of participants	149	131	124	151
No. missing	1	0	0	1
Score (95% CI)	3.04 (2.63-3.45)	3.81 (3.34-4.28)	3.93 (3.46-4.39)	3.49 (3.06-3.91)
Wave 2				
No. of participants	133	115	117	141
No. missing	0	1	0	0
Score (95% CI)	3.00 (2.57-3.43)	3.59 (3.09-4.09)	4.22 (3.76-4.69)^b^^,^^c^	4.30 (3.87-4.74)^b^^,^^c^

^a^ Group 1 received no messages; group 2, a message from a clinician only in wave 1; group 3, messages from a news story in wave 1 and a clinician in wave 2; and group 4, messages from a family member in wave 1 and a clinician in wave 2. Both outcomes were measured on a 7-point scale. Higher scores indicate higher support for stopping screening in older women (1 indicates definitely should get a mammogram; 7, definitely should not get a mammogram) and higher intention to stop screening for oneself (1 indicates very likely to get a mammogram; 7, very unlikely to get a mammogram). Comparisons among the experimental groups at wave 2 used analysis of variance with the Tukey test. There was no significant difference between groups 3 and 4 for any of the comparisons.

^b^ The mean was significantly different when compared with group 1 (*P* ≤ .05).

^c^ The mean was significantly different than group 2 (*P* ≤ .05).

**Figure 2.**
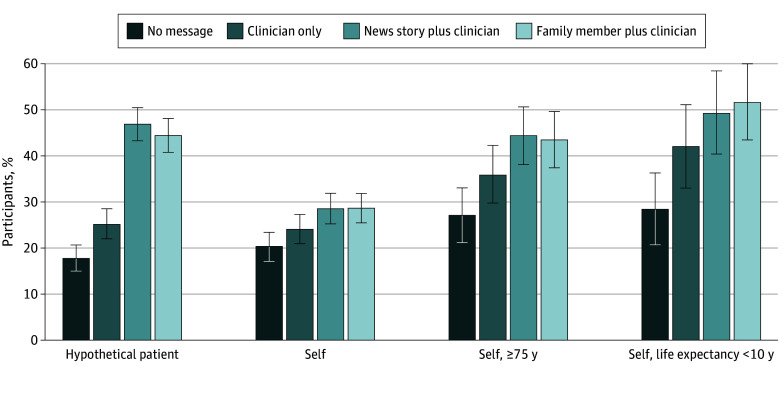
Percentages of Participants Who “Supported Screening Cessation” at Wave 2 Regarding the Hypothetical Patient and Themselves Support for screening cessation was defined as scores 5 to 7 on the 7-point scale when asked about whether the hypothetical patient should stop screening or about one’s own screening cessation intentions in the next 2 years. Error bars indicate 95% CIs.

At wave 2, group 1 again had the lowest support for stopping screening for the hypothetical patient (mean score, 2.68 [95% CI, 2.54-2.82]) (Table 2). In group 2, which received a single message from a clinician at wave 1 and no message at wave 2, the message effect attenuated over time, but support for stopping screening was still significantly higher than group 1, with mean score of 3.14 (95% CI, 2.99-3.29; *P* < .001). Groups 3 and 4, which received 2 stopping screening messages over time, had the highest support for screening cessation; the mean scores were 4.23 (95% CI, 4.09-4.38) for the news story plus clinician message (group 3) and 4.12 (95% CI, 3.97-4.27) for the family member plus clinician messages (group 4). These 2 scores were significantly higher compared with groups 1 and 2 (*P* < .001 for all comparisons) but were not significantly different from each other (*P* = .70). When the outcome was dichotomized, this translated to an increase from 121 participants (17.8%) who supported screening cessation for the hypothetical patient in group 1 to 331 (47.0%) among participants in group 3 (Figure 2).

### Screening Cessation Intention for Oneself

Messaging effects on screening cessation intentions for oneself followed a similar pattern as that on support for screening cessation for the hypothetical patient, but the magnitude of the effects was smaller. At wave 2, group 1 had the lowest intention to stop screening (mean score, 2.38 [95% CI, 2.21-2.55]) (Table 2). Group 2, with a single clinician-delivered message, had higher intentions to stop screening but was not significantly different than group 1 (mean score, 2.59 [95% CI, 2.42-2.77]; *P* = .35). The cumulative effects of messages from 2 sources resulted in significantly higher intentions to stop screening than groups 1 and 2 (all *P* < .05); mean scores were 2.95 (95% CI, 2.77-3.13) for group 3 and 2.98 (95% CI, 2.80-3.17) for group 4.

Participants who were 75 years or older or with a life expectancy of less than 10 years had higher intentions to stop screening and larger message effects compared with the entire sample (Table 2 and Figure 2). Among participants 75 years or older, intentions to stop screening at wave 2 had a mean score of 3.83 (95% CI, 3.50-4.16) for group 3 and 3.80 (95% CI, 3.46-4.13) for group 4; these were significantly higher compared with group 1 (2.82 [95% CI, 2.49-3.16]; *P* < .001 for both). Among participants with a life expectancy of less than 10 years, intention to stop screening at wave 2 had a mean score 4.22 (95% CI, 3.76-4.69) for group 3 and 4.30 (95% CI, 3.87-4.74) for group 4; again, these scores were significantly higher than those for group 1 (3.00 [95% CI, 2.57-3.43]; *P* < .001 for both). Participants with high breast cancer risk had lower intentions to stop screening (eTable 3 in Supplement 2). The only significant message effect within this group was that group 4 had higher intentions to stop screening (2.96 [95% CI, 2.56-3.36]) compared with group 1 (1.97 [95% CI, 1.64-2.30]; *P* = .009), suggesting the importance of the information source from family members among older women at higher breast cancer risk.

### Other Consequences of Message Exposure

Participants reported low rates of negative perceptions of mammograms after reading the message. All mean scores were less than 3 (the neutral option on the 5-point scale) regarding whether the message discouraged one from getting mammograms, made mammograms seem unpleasant, and made one concerned about mammograms (Table 3), with only 3.5% (27 of 763) to 10.2% (77 of 756) who strongly agreed with any statement across groups. Similarly, we found low rates of reported worry or annoyance after reading the message (all means <3), with only 4.0% (30 of 754) to 8.3% (63 of 759) who felt extremely worried and 11.8% (89 of 752) to 14.1% (106 of 754) who felt extremely annoyed across groups.

**Table 3.** Means Scores for Other Consequences From Message Exposure^a^

Consequence of exposure	Participant group			
	Group 1 (n = 756)	Group 2 (n = 761)	Group 3 (n = 762)	Group 4 (n = 772)
Negative perceptions of mammograms^b^				
This information discourages me from wanting to get a mammogram	NA	2.34 (2.25-2.42)	2.29 (2.20-2.38)	2.12 (2.03-2.20)
No. missing	NA	6	7	9
This information makes getting a mammogram seem unpleasant to me	NA	2.50 (2.41-2.59)	2.55 (2.46-2.64)	2.39 (2.30-2.48)
No. missing	NA	6	9	6
This information makes me concerned about the health effects of getting a mammogram	NA	2.78 (2.69-2.88)	2.74 (2.65-2.83)	2.47 (2.38-2.56)
No. missing	NA	5	5	6
Negative emotional responses^c^				
Worried	NA	2.42 (2.33-2.51)	2.15 (2.07-2.24)	2.26 (2.16-2.35)
No. missing	NA	12	8	13
Annoyed	NA	2.52 (2.42-2.62)	2.54 (2.44-2.64)	2.66 (2.55-2.76)
No. missing	NA	9	10	18

Abbreviation: NA, not applicable.

^a^ Data are presented as mean (95% CI) unless noted otherwise. Outcomes were only assessed at wave 1. Group 1 received no messages; group 2, a message from a clinician only in wave 1; group 3, messages from a news story in wave 1 and a clinician in wave 2; and group 4, messages from a family member in wave 1 and a clinician in wave 2.

^b^ Adapted from scale used in health communication.^27^ Measured on 5-point Likert scale where 1 indicates strongly disagree; 5, strongly agree.

^c^ Measured on 5-point Likert scale where 1 indicates not at all; 5, extremely.

## Discussion

This is the first national study, to our knowledge, to rigorously test the effects of messaging about breast cancer screening cessation. We found that message exposure increased support for and intentions of stopping screening among older women for whom stopping screening would be appropriate based on age (≥75 years) or health (life expectancy of <10 years), and the effect was greatest when the message was delivered from multiple sources. Although messaging strategies have been used to change other health behaviors,^17,18,32^ they have not been used to encourage screening cessation to reduce overscreening in older women. These results show that messaging is a viable and potentially effective approach for reducing overscreening.

For a hypothetical older woman with multimorbidity and functional impairment, message exposure increased support for stopping screening by almost 30% (1.4-1.6 points on a 7-point scale). This was achieved with low rates of negative perception of mammograms or negative emotional response. The message effect was appropriately small on participants’ own screening intentions since most participants did not meet guideline criteria for stopping screening. The message effect was also appropriately small among participants with higher cancer risk. In the subgroups of women who met guideline criteria for stopping screening, the message effect was larger. Among those 75 years or older or with life expectancy of less than 10 years, the increase in intention to stop screening was 1.0 to 1.2 points on a 7-point scale. This is a sizeable change; in a randomized clinical trial of a breast cancer decision aid among older women, each 1-point change on a 15-point scale of screening intention corresponded with a 10% decrease in screening rate^33^; a 1-point change on a 7-point scale may potentially correspond to a 10% or larger decrease in screening rate but needs to be confirmed in future studies that measure screening behavior. Although these analyses were exploratory, our findings suggest that messaging holds promise to selectively reduce screening in older women for whom it would be appropriate to stop screening without significantly reducing screening in older women who may still benefit.

Our study of the cumulative effects from multiple message exposures on screening was novel. Literature on individualized cancer screening for older adults highlights the importance of multilevel influences.^34^ Our findings suggest that while clinicians are an important target for efforts to reduce overscreening, leveraging other information sources may increase impact. Although we found low levels of negative reactions to the message, engaging key partners to devise message delivery can help identify strategies to further minimize undesirable outcomes from messaging.

### Strengths and Limitations

Strengths of our study include a large national sample, randomized experimental design, and study of message exposure over time. Limitations include sampling bias and nonresponse bias. Compared with national census data,^35^ our study participants were younger, more likely to be White, and had a higher level of education. We only included English-speaking women, and the rate of low health literacy was lower than in some other national studies.^36,37,38^ These differences may affect the generalizability of our findings, but the randomized design helps ensure internal validity. Future studies need to examine whether our results can be replicated in different populations. Our primary outcome focused on a hypothetical scenario where the responses may not fully reflect perspectives in real clinical decisions; we mitigated this by also assessing self-screening intentions. We focused on studying message sources in addition to the clinician and did not include combinations of nonclinician sources. Our recruitment exceeded the planned sample size, and the added statistical power can reveal nonmeaningful differences. However, we followed our analysis plan formulated a priori, and our results have face validity. Last, our study design did not allow assessment of actual change in screening behavior. However, screening intention is a strong predictor of behavior.^39^

## Conclusions

In this randomized 2-wave clinical trial, we demonstrate that a message describing the rationale for stopping screening can significantly increase support and intention for appropriate screening cessation among older women. This message can help inform public- or patient-facing materials and be incorporated into decision-support tools and electronic medical record prompts to optimize screening. The message can also be delivered as a stand-alone intervention to reduce overscreening. Future work should engage potential message sources to examine the feasibility and acceptability of multilevel messaging strategies and their effect on screening behavior.

## Notes

Supplement 1.
Trial Protocol

Supplement 2. **eTable 1.** Comparison of Responders vs Nonresponders **eTable 2.** Comparison of Participants Who Completed Both Waves 1 and 2 vs Those Who Completed Wave 1 Only and Did Not Complete Wave 2 **eTable 3.** Mean Outcome Scores Regarding Stopping Screening Intention for Oneself Among Those With Higher Breast Cancer Risk **eAppendix.** Survey Instrument

Supplement 3.
Data Sharing Statement
