# Supplementary material for: Effect of Messaging on Support for Breast Cancer Screening Cessation Among Older US Women: A Randomized Clinical Trial
Source: JAMA Netw Open. 2024 Aug 19;7(8):e2428700. doi: 10.1001/jamanetworkopen.2024.28700 (PMC11333986; doi:10.1001/jamanetworkopen.2024.28700)
Supplement: Supplement 1. — Trial Protocol [file jamanetwopen-e2428700-s001.pdf]

Study Protocol (This protocol followed the SPIRIT guideline)

Title: Messaging strategies to reduce breast cancer over-screening in older women

Protocol date: 4/4/2023

Authors: Nancy L. Schoenborn, MD, MHS;[1]
Contributors: Sarah E. Gollust, PhD,[2] Rebekah H. Nagler, PhD,[3] Craig E. Pollack, MD, MHS,[4] Cynthia M. Boyd, MD, MPH,[1,4,5] Qian-Li Xue, PhD,[1,5] Mara A. Schonberg, MD, MPH,[6]

1. Johns Hopkins University School of Medicine, Department of Medicine, Division of Geriatric Medicine and Gerontology.
2. University of Minnesota School of Public Health, Division of Health Policy and Management.
3. University of Minnesota Hubbard School of Journalism and Mass Communication.
4. Johns Hopkins University Bloomberg School of Public Health, Department of Health Policy and Management.
5. Johns Hopkins Center on Aging and Health
6. Beth Israel Deaconess Medical Center, Harvard Medical School, Division of General Medicine and Primary Care

Conflict of interest: No author or contributor has any conflicts of interests related to the study.

A. Trial registration:

Primary registry and ID	ClinicalTrials.gov NCT05821023
Source of funding	NIA R01AG066741
Sponsor	Johns Hopkins University
Human subjects review	IRB00283392
Contact	Nancy Schoenborn MD; nancyschoenborn@jhmi.edu; 4105507142
Study description	This is an online survey experiment with data collection over 2 time points two weeks apart. This is Aim 2 of a three-aim R01 project; overall project goal is to better understand how messages from different sources interact to affect older women's breast cancer screening decisions. In this current project, the investigators propose to test the effect of combined exposure to a clinician message + a message from another source (i.e. family/friend or media) on older women's breast cancer screening beliefs, attitudes, and intentions.
Health conditions	Breast cancer screening
Study design	Randomized controlled interventional study with parallel assignment to 1 of 6 groups
Interventions	Group 1 – no message exposure;<br>Group 2 – single clinician message at Wave 1 on screening cessation; no message at Wave 2.

	Group 3 – message from news story at Wave 1 on screening cessation; message from clinician at Wave 2 on screening cessation.<br>Group 4 – message from family member at Wave 1 on screening cessation; message from clinician at Wave 2 on screening cessation.<br>Group 5 – message from news story at Wave 1 on screening continuation; message from clinician at Wave 2 on screening cessation.<br>Group 6 – message from family member at Wave 1 on screening continuation; message from clinician at Wave 2 on screening cessation.
Outcome measures	Primary outcomes: support for stopping screening for a hypothetical older woman in the survey vignette<br>Secondary or other pre-specified outcomes: screening intention for participants; breast cancer screening attitudes; perceived message effectiveness; emotional response; negative perceptions of mammograms; awareness of screening harms; confusion or backlash about conflicting messages
Eligibility	Inclusion criteria:65 years; female; part of an online survey panel called KnowledgePanel; able to complete survey in English.<br>Exclusion criteria: personal history of breast cancer
Study start date	May 12, 2023
Study completion date	June 19, 2023

B. Background and Objectives (Specific Aims)

Breast cancer is the most common life-threatening cancer in women.[1,2] Mammography screening may decrease cancer-related mortality and morbidity, but the potential benefits of screening are often delayed for many years while significant harms, such as over-diagnosis and over-treatment of clinically unimportant cancers, can occur in the short-term.[3,4-14] Despite guidelines recommending against routine breast cancer screening in older women with limited life expectancies,[14-20] many of these older women continue to be screened.[21,22,23] The overarching premise of this project is that clinician-patient communication holds promise to promote appropriate screening cessation and reduce over-screening in older women but its impact may be weakened by conflicting messages from other sources. Better understanding the interplay between messages from clinical and non-clinical sources is crucial to inform interventions.

Clinicians are an important and trusted source of information regarding cancer screening and their recommendations strongly influence patients' screening behavior.[21,24-28] Our prior research suggests that a) clinicians are increasingly willing to recommend that older patients forgo screening [29-31] and b) older patients are increasingly willing to accept these recommendations in the setting of a trusting relationship.[31,32] However, clinicians reported barriers to stopping routine cancer screening: one barrier in particular is that patients often receive contradictory (and largely pro-screening) messages from their family, friends, or the media.[33-35]

Indeed, clinician-patient communication does not occur in a vacuum but rather happens in the setting of patients' social relationships and the broader media landscape.[24-26,33-35] Prior research found that experiences with friends or family members who had screening-detected

early stage breast cancers were associated with significant increase in women's own intentions to get screened (Odds Ratio 2.89).[36] Also, media coverage has often focused on screening's benefits and screening initiation rather than the decision about stopping screening.[37-42] For example, we found that among televised news stories reporting on the American Cancer Society's 2015 breast cancer screening recommendations, 94.4% reported on when screening should start, but only 3.4% reported that life expectancy should be used to decide whether or not to stop screening.[37]

In this context, even when clinicians are willing and able to discuss screening cessation with older women, the impact of their messages may be reduced or negated altogether by competing information. Conversely, if messages from the media and patients' social networks (i.e. family/friends) were to align with that from the clinicians, they could have a powerful synergistic influence to reduce over-screening. Before an effective messaging intervention can be developed to reduce over-screening, it is critical to first identity which messages/messengers are likely to have the greatest impact. <u>This project addresses these knowledge gaps by examining how messages from clinicians, social networks, and media, separately and together, impact older women's screening intentions, with the goal of informing a messaging intervention to reduce over-screening.</u>

(Prior work) Aim 1 – To establish which messages intended to reduce breast cancer over-screening are considered most credible among older women. Establishing message credibility is important before assessing message impact since messages that are not credible are more likely to be rejected.[43-45] In a national online survey, we will randomize 600 older women (age 65+ years) to read and evaluate the credibility of different types of messages (e.g. evidence, anecdote) that describe the harms of over-screening. We will systematically vary the information source - clinician, family/friend, or media - for each message.

(Current project) Aim 2 – To identify the combined effects of consistent and conflicting messages from different sources—including clinicians, social relationships, and media—on breast cancer screening beliefs, attitudes, and intentions over time. Using top-rated messages from Aim 1, we will conduct a two-wave online survey experiment with 3000 older women from a nationally representative panel. The two waves will include a clinician message intended to reduce over-screening (i.e. mentions harms of over-screening, supports screening cessation) and a non-clinician message. We will vary whether the non-clinician message is from family/friend or the media; we will also vary whether the non-clinician message supports or opposes screening cessation. We hypothesize that the effect of clinician message on screening intentions will be strengthened when paired with consistent messages and weakened when paired with conflicting messages.

(Next step) Aim 3 – To engage multi-disciplinary stakeholders to devise message delivery strategy for subsequent intervention. We will conduct focus groups with 30-40 stakeholders representing the end users of the messaging strategies, including clinicians, health system administrators, older women, caregivers, community leaders, advocacy group representatives, and health journalists. Focus groups will help devise strategies to deliver, in a real-world context, the messages from Aim 2 that most effectively reduced screening intentions.

This innovative work will produce much needed data on effective strategies to communicate the rationale for stopping cancer screening when harms outweigh the benefits. The results will directly inform a novel communication intervention, as next step, to reduce breast cancer over-screening among older women.

C. Description of study design

C.1. Trial Design: Two-wave randomized controlled online survey experiment.

C.2. Study Setting / Recruitment / Randomization / Blinding / Data collection

We will recruit participants from the KnowledgePanel, which is a nationally representative online survey panel maintained by a survey research company Ipsos. KnowledgePanel is a largest probability-based online panel consisting of about 55,000 adult members that covers 97% of the U.S. adult population.[46] KnowledgePanel includes good representation of older women across the spectrum of different health status. Panel members are randomly recruited by random digit dialing telephone (until 2009) or by address (since 2009); the sampling frame includes both listed and unlisted numbers and households are provided with access to internet and hardware if needed.[46,47] Extensive analyses by independent researchers have consistently found the KnowledgePanel to closely match other nationally representative surveys such as the Health and Retirement Study.[48,49] KnowledgePanel has been used in numerous medical research studies involving older adults, including our own prior work.[50-53] Based on prior experience we expect a response rates of 65-70% among older adults who are invited to participate.

KnowledgePanel will be responsible for inviting panel members to the study via email and for programming the survey for online administration. During the survey programming, randomization will be achieved using randomly generated numbers. To ensure matched participant characteristics across groups, participants will be sorted by the randomly generated numbers and by race/ethnicity, geographic region, education (these information have already been collected by KnowledgePanel about its members), then the participants, thus sorted, will be allocated sequentially to each of the 6 experimental groups. Participants and the research team will be blinded to the allocation until data collection is complete. Only de-identified data will be sent from KnowledgePanel to the study team.

KnowledgePanel will pretest each wave of the survey with individuals not included in the study to ensure programming and randomization procedures occur as intended. Specifically, KnowledgePanel will pretest wave 1 survey with 50 eligible older women, and pretest wave 2 survey with the same individuals, recognizing that not all 50 may complete wave 2's pretest.

C.3. Eligibility Criteria

- Inclusion criteria: 65 years; female; part of KnowledgePanel; able to complete survey in English.
- Exclusion criteria: personal history of breast cancer

KnowledgePanel already collects information on panel members' age, gender, English proficiency. We will independently include a screening question at the beginning of the survey regarding breast cancer history.

C.4. Interventions and Choice of comparator:

We will randomly assign women meeting eligibility to 6 groups (Table 1). The experimental groups 3-6 will read a message at Wave 1 that may be from either family/friend or the media followed by a second message from a clinician one to two weeks later at Wave 2, as research suggests that message decay occurs in 1-2 weeks.[54] By presenting the non-clinician message first, we aim to mimic the real-world scenario where patients are often exposed to information from their family, friends and/or the media before a clinic visit.

The clinician message will be directed at reducing over-screening, mentioning the harms of over-screening and supporting screening cessation. We will systematically vary the non-

clinician message to be either consistent with the clinician message (also mentions harms of over-screening and supports screening cessation) or conflicting (mentions benefits of screening, supports continued screening and opposes screening cessation). We include a no-exposure control group (Group 1) where the participants read no message at either time point. We also include a single exposure group that reads only the clinician message (Group 2).

Table 1. Aim 2 experimental design

Group #	Group type	Wave 1		Wave 2	
		Source	Content	Source	Content
1	Control (no exposure)	No message	No message	No message	No message
2	Control (single exposure)	Clinician	Stop screening	No message	No message
3	Experimental	Media	Stop screening	Clinician	Stop screening
4	Experimental	Family/Friend	Stop screening	Clinician	Stop screening
5	Experimental	Media	Continue screening	Clinician	Stop screening
6	Experimental	Family/Friend	Continue screening	Clinician	Stop screening

C.5. Outcomes and timeline (Table 2)**:**

Table 2. Aim 2 study outcomes and timing of assessments.

	Outcomes	Group 1		Group 2		Group 3/4		Group 5/6	
		W1	W2	W1	W2	W1	W2	W1	W2
Primary	Support for screening cessation for hypothetical patient	x	x	x	x	x	x	x	x
Secondary	Screening intention for oneself	x	x	x	x	x	x	x	x
Secondary	Screening attitudes	x	x	x	x	x	x	x	x
Secondary	Awareness of harms of screening	x		x		x		x	
Secondary	Negative perceptions of mammograms			x		x		x	
Secondary	Perceived effects on making one think more carefully or seek more information about mammograms			x		x	x	x	x
Secondary	Emotional response			x		x		x	
Exploratory	Outcomes related to conflicting messages (confusion, backlash, ambivalence)	x					x		x
Exploratory	Open-ended impression			x		x		x	

<u>Support for screening cessation for hypothetical patient:</u> "How strongly do you believe that older women with a lot of health problems like <u>Ms. Johnson</u> should get a mammogram in the next two years? Measured on a 7-point scale (1=definitely should get a mammogram; 7= definitely should not get a mammogram).

<u>Screening intention for oneself: "</u>How likely is it that <u>you</u> will get a mammogram in the next two years?" Measured on a 7-point scale (1=very likely to get a mammogram, 7=very unlikely to get a mammogram).[55]

<u>Attitudes:[55]</u>

- "For <u>you</u>, would you say getting a mammogram in the next two years is…1=unimportant; 5=important; 1=harmful; 5=beneficial; 1=not worthwhile; 5=worthwhile;

- "For older women with a lot of health problems like Ms. Johnson, would you say getting a mammogram in the next two years is…1=unimportant; 5=important; 1=harmful; 5=beneficial; 1=not worthwhile; 5=worthwhile;

Awareness of harms of screening: "Have you ever heard or read the following information? You could have heard or read this information during this survey or previously." (Yes/No)

- For some women over age 65 who have a lot of health problems, medical guidelines recommend against regular mammograms.
- Mammograms can find slow-growing breast cancers that might never cause problems but can lead to unnecessary, risky treatments such as surgery or radiation.
- Mammograms can have false alarm results that may cause stress, anxiety, and require breast biopsy.

Negative perceptions of mammograms:[56,57]

"Thinking about the information that was shared, please rate how much you agree or disagree with the following statement." (1=strongly disagree; 5=strongly agree)

- This information discourages me from wanting to get a mammogram.
- This information makes getting a mammogram seem unpleasant to me.
- This information makes me concerned about the health effects of getting a mammogram.

Effect perceptions:

"Thinking about the information that was shared, please rate how much you agree or disagree with the following statement." (1=strongly disagree; 5=strongly agree)

- This information would make me think carefully about getting a mammogram
- This information would make me want to find out more information about the potential benefits of getting a mammogram.
- This information would make me want to find out more information about the potential downsides of getting a mammogram.

Emotional response:[58]

"The next question includes words that describe different feelings and emotions. Please indicate how you feel after reading the information that was shared". (1=not at all; 5=extremely)

- Annoyed
- Interested
- Worried
- Reassured

Outcomes related to conflicting messages:[59]

"Please rate how much you agree or disagree with the following statement." (1=strongly disagree; 5=strongly agree)

- I find mammogram screening recommendations to be confusing.
- It is clear to me whether I should continue or stop getting mammograms.
- Medical guidelines provide good advice about mammogram screening.
- I trust my doctors' advice about whether I should continue or stop getting mammograms.
- I have mixed feelings about getting mammograms.

Open-ended impressions: "We are now interested in what you were thinking about when you read the information that was shared. Please use the text box below to record your thoughts and ideas. Don't worry about spelling and grammar. Please be completely honest and list all of the thoughts that you had."

C.6. Statistical methods and sample size.

The main analysis focuses on the primary outcome assessed at Wave 2 with the independent variables being the randomly assigned message/messenger combinations. The primary goal is to compare the experimental groups (Groups 3-6) at Wave 2 with both control groups to assess the combined effect of consistent and conflicting messages over time. We hypothesize that the participants who read consistent messages both directed at reducing over-screening (Groups 3,4) will have higher support and higher intentions to stop screening than participants who read clinician message alone (Group 2); the participants who read conflicting messages (Groups 5,6) will have lower intentions to stop screening than participants who read clinician message alone (Group 2). We will compare the mean scores across groups using ANOVA and adjust for multiple comparisons. We will conduct exploratory analyses by key covariates, including age, predicted life expectancy based on health and functional status, to examine the impact of messaging within these subgroups.

Power calculations are based on preliminary data in which we asked 41 patients 65+ about willingness to stop routine cancer screening if recommended by their clinician. Based on the distribution of responses from this preliminary data, we estimate that with 400 participants randomized to each group at Wave 2, we will have 80% power to detect a 0.30 point difference for the primary outcome. The survey company Ipsos that manages and maintains the KnowledgePanel affirms that, through their recruiting strategy and based on their past experience, 20% attrition or less is expected between the two waves over 1-2 weeks. Assuming 20% attrition, we will target 500 per group at Wave 1.

D. Protection of human subjects

D.1. Consent or assent

We will include a sentence at the beginning of the survey that completion of the survey serves as consent to participate in the study..

D.2. Potential risks and harms

The study poses very little risk to participants. Only de-identified data will be provided to the study team by KnowledgePanel and only de-identified data will be used during data analysis in order to protect confidentiality and privacy of the participants. There is burden of taking the time to participate in the study. We estimate that it will take approximately 15 minutes to complete the survey at time 1 and 10 minutes at time 2. In addition, it is possible that some participants may develop feelings of anxiety, distress, or fatigue when answering survey questions. We will state at the beginning of the survey that participants may pause or stop at any time, skip any questions that make them uncomfortable, and/or withdraw their participation.

D. 3. Protection Against Risks

The utmost respect will be paid to participant privacy. All materials obtained will be used specifically for research purposes and will be kept confidential; they will be stored on secure

drivers where the files will be password-protected, or in locked file cabinets in the office of an investigator; the offices have locked doors and are in buildings where ID cards are required for entry. All participant information will not be shared with anyone other than those working on this study. Each participant will be given a unique study ID, only de-identified information will be provided by KnowledgePanel to the study team and only de-identified data will be used during data analysis in order to protect confidentiality and privacy of the participants. All study investigators will be trained in HIPAA guidelines and requirements and in human subject protection and in Good Clinical Practice (GCP). At the earliest opportunity and in compliance with NIH policy, any printed data will be shredded and disposed of in locked recycle bins, computer files will be deleted.

D.4. Potential Benefits of the Proposed Research to Research Participants and Others

There is no personal benefit to the participants although they may be gratified to share their perspectives on the study topic and/or contribute to the research goal to improve cancer screening in older women. Ipsos maintains the KnowledgePanel by providing both survey-specific and non-survey-specific incentives using a point system that the participants can then redeem for cash, gift cards, merchandise, or game entries.

There is significant benefit to society in that the information gathered by the study team will help move the field forward in understanding what messaging strategies are most effective in reducing breast cancer over-screening among older women. This information is not currently known and is both necessary and critical to inform interventions to improve cancer screening practices and the care of older women. The risks from this study are minimal while the potential benefits are large. Therefore, we believe that the minimal risks to subjects are reasonable.

D.5. Importance of the Knowledge to be Gained

This study will allow for better understanding of how consistent and conflicting messages from different sources – including clinicians, social relationships, and new media – affect breast cancer beliefs, attitudes, and intentions among older women. The results are important to inform a communication intervention to reduce breast cancer over-screening and promote more informed decision making about breast cancer screening in older women.

E. Dissemination of research results

We will disseminate the research results by presentations at national and international meetings and conventional publications in the medical literature. Full results of the study will be published as soon as possible, consistent with principles of peer-reviewed publication. National meetings of the American Geriatrics Society, Society of General Internal Medicine, American Society of Clinical Oncology, and Society of Medical Decision Making are excellent venues for presenting study results.

F. **Ethical approval** (see addend IRB approval letter at the end of the protocol)

References:

1. SEER Stat Factsheets: Breast Cancer. Bethesda, MD: National Cancer Institute; 2015. http://seer.cancer.gov/statfacts/html/breast.html, accessed on June 2020
2. American Cancer Society. Breast Cancer Facts & Figures 2017-2018. Atlanta: American Cancer Society, Inc. 2017.
3. Walter LC, Schonberg MA. Screening mammography in older women: A review. *JAMA*. 2014;311(13):1336-1347.
4. Pace LE, Keating NL. A systematic assessment of benefits and risks to guide breast cancer screening decisions. *JAMA* 2014;311:1327-35.
5. Nystrom L, Andersson I, Bjurstam N, Frisell J, Nordenskjold B, Rutqvist LE. Long-term effects of mammography screening: updated overview of the Swedish randomised trials. *Lancet* 2002;359:909-19.
6. Lee SJ, Boscardin WJ, Stijacic-Cenzer I, Conell-Price J, O'Brien S, Walter LC. Time lag to benefit after screening for breast and colorectal cancer: Meta-analysis of survival data from the United States, Sweden, United Kingdom, and Denmark. *BMJ*. 2013;346:e8441.
7. Walter LC, Covinsky KE. Cancer screening in elderly patients: A framework for individualized decision making. *JAMA*. 2001;285(21):2750-2756.
8. Morris E, Feig SA, Drexler M, Lehman C. Implications of overdiagnosis: impact on screening mammography practices. *Popul Health Manag.* 2015;18 Suppl 1: S3-11.
9. Lansdorp-Vogelaar I, Gulati R, Mariotto AB, et al. Personalizing age of cancer screening cessation based on comorbid conditions: Model estimates of harms and benefits. *Ann Intern Med*. 2014;161(2):104-112.
10. Siu AL, U.S. Preventive Services Task Force. Screening for breast cancer: U.S. Preventive Services Task Force recommendation statement. *Ann Intern Med.* 2016;164(4):279-96.
11. Eckstrom E, Feeny DH, Walter LC, Perdue LA, Whitlock EP. Individualizing cancer screening in older adults: A narrative review and framework for future research. *J Gen Intern Med*. 2013;28(2):292-298.
12. Soung MC. Screening for cancer: when to stop?: A practical guide and review of the evidence. *Med Clin North Am.* 2015 Mar;99(2):249-62.
13. Saini SD, van Hees F, Vijan S. Smarter screening for cancer: Possibilities and challenges of personalization. *JAMA*. 2014;312(21):2211-2212.
14. Harris RP, Wilt TJ, Qaseem A, High Value Care Task Force of the American College of Physicians. A value framework for cancer screening: Advice for high-value care from the American College of Physicians. *Ann Intern Med*. 2015;162(10):712-717.
15. Oeffinger KC, Fontham ET, Etzioni R, et al. Breast cancer screening for women at average risk: 2015 guideline update from the American Cancer Society. *JAMA.* 2015;314(15):1599-614.
16. Bevers TB, Helvie M, Bonaccio E, et al. NCCN Guidelines. Breast Cancer Screening and Diagnosis (Version 3.2018). https://www2.tri-kobe.org/nccn/guideline/breast/english/breast-screening.pdf, accessed June 2020.

17. Society of General Internal Medicine. Five things physicians and patients should question - the choosing wisely campaign. 2013. http://www.choosingwisely.org/wp-content/uploads/2013/09/SGIM-5things-List_091213.pdf, accessed June 2020.

18. AGS Choosing Wisely Workgroup. American geriatrics society identifies another five things that healthcare providers and patients should question. *J Am Geriatr Soc*. 2014;62(5):950-960.

19. Williams AW, Dwyer AC, Eddy AA, et al. Critical and honest conversations: The evidence behind the "choosing wisely" campaign recommendations by the American Society of Nephrology. *Clin J Am Soc Nephrol*. 2012;7(10):1664-1672.

20. The Society for Post-Acute and Long-Term Care Medicine. Ten things physicians and patients should question. AMDA Choosing Wisely List 2015. http://www.choosingwisely.org/wp-content/uploads/2015/02/AMDA-Choosing-Wisely-List.pdf, accessed June 2020.

21. Schonberg MA, Breslau ES, McCarthy EP. Targeting of mammography screening according to life expectancy in women aged 75 and older. *J Am Geriatr Soc*. 2013;61(3):388-395

22. Royce TJ, Hendrix LH, Stokes WA, Allen IM, Chen RC. Cancer screening rates in individuals with different life expectancies. *JAMA Intern Med*. 2014 Oct;174(10):1558-65.

23. Schoenborn NL, Huang J, Sheehan OC, Wolff JL, Roth DL, Boyd CM. Influence of age, health and function on cancer screening in older adults with limited life expectancy. *J Gen Intern Med.* 2019;34(1):1100-117.

24. Schonberg MA, McCarthy EP, York M, Davis RB, Marcantonio ER. Factors influencing elderly women's mammography screening decisions: implications for counseling. *BMC Geriatr.* 2007;7:26.

25. Sutkowi-Hemstreet A, Vu M, Harris R, Brewer NT, Dolor RJ, Sheridan SL. Adult patients' perspectives on the benefits and harms of overused screening tests: a qualitative study. *J Gen Intern Med.* 2015;30(11):1518-26.

26. Hoffman RM, Lewis CL, Pignone MP, et al. Decision-making processes for breast, colorectal, and prostate cancer screening: the DECISIONS survey. *Med Decis Making.* 2010;30(5 Suppl): 53S-64S.

27. Haas JS, Barlow WE, Schapira MM, et al. Primary Care Providers' Beliefs and Recommendations and Use of Screening Mammography by their Patients. *J Gen Intern Med.* 2017;32(4): 449-457.

28. Tan A, Kuo YF, Goodwin JS. Potential overuse of screening mammography and its association with access to primary care. *Med Care.* 2014;52(6): 490-5.

29. Schoenborn NL, Bowman TL, Cayea D, Pollack CE, Feeser S, Boyd C. Primary care practitioners' views on incorporating long-term prognosis in the care of older adults. *JAMA Intern Med*. 2016 May 1;176(5):671-8.

30. Schoenborn NL, Bowman TL, Cayea D, Boyd C, Feeser S, Pollack CE. Discussion strategies that primary care clinicians use when stopping cancer screening in older adults. *J Am Geriatr Soc*. 2016 Nov;64(11):e221-e223.

31. Schoenborn NL**,** Boyd C, Lee S, Cayea D, Pollack CE. Communicating about stopping cancer screening: comparing clinicians' and older adults' perspectives. *Gerontologist*. 2019;59(Suppl 1):S67-S76.

32. Schoenborn NL, Lee K, Pollack CE, Armacost K, Dy S, Bridges JF, Xue QL, Wolff A, Boyd C. Older adults' views and communication preferences about cancer screening cessation *JAMA Intern Med*. 2017;177(8): 1121-1128.

33. Henriksen MJ, Guassora AD, Brodersen J. Preconceptions influence women's perceptions of information on breast cancer screening: a qualitative study. *BMC Res Notes.* 2015;8:404.

34. He X, Schifferdecker KE, Ozanne Em, Tosteson ANA, Woloshin S, Schwartz LM. How do women view risk-based mammography screening? A qualitative study. *J Gen Intern Med.* 2018;33(11):1905-1912.

35. Schoenborn NL, Massare J, Park R, Boyd CM, Choi Y, Pollack CE. Assessment of clinician decision-making on cancer screening cessation in older adults with limited life expectancy. *JAMA Network Open*. 2020 Jun 1;3(6):e206772.

36. Nowak SA, Parker AM. Social network effects of nonlifesaving early-stage breast cancer detection on mammography rates. *Am J Public Health.* 2014;104(12):2439-44.

37. Nagler RH, Fowler EF, Marino NM, Mentzer KM, Gollust SE. The evolution of mammography controversy in the news media: a content analysis of four publicized screening recommendations, 2009 to 2016. *Womens Health Issues.* 2019;29(1):87-95.

38. Schwartz LM, Woloshin S. News media coverage of screening mammography for women in their 40s and tamoxifen for primary prevention of breast cancer. *JAMA.* 2002;287(23):3136-42.

39. Smith KC, Kromm EE, Klassen AC. Print news coverage of cancer: what prevention messages are conveyed when screening is newsworthy? *Cancer Epidemiol.* 2010;34(4):434-41.

40. Katz ML, Sheridan S, Pignone M, et al. Prostate and colon cancer screening messages in popular magazines. *J Gen Intern Med.* 2004;19(8):843-8.

41. MacKenzie R, Chapman S, Barratt A, Holding S. "The news is [not] all good": misrepresentations and inaccuracies in Australian news media reports on prostate cancer screening. *Med J Aust.* 2007;187(9):507-10.

42. Cooper CP, Gelb CA, Hawkins NA. How many "get screened" messages does it take? Evidence from colorectal cancer screening promotion in the United States, 2012. *Prev Med.* 2014;60:27-32.

43. Pornpitakpan C. The persuasiveness of source credibility: A critical review of five decades' evidence. *J Appl Soc Psychol.* 2004;34(2):243-281.

44. Fiske ST, Dupree C. Gaining trust as well as respect in communicating to motivated audiences about science topics. *Proc Natl Acad Sci U S A.* 2014;111 Suppl 4:13593-7.

45. Zhao X, Strasser A, Cappella JN, Lerman C, Fishbein M. A measure of perceived argument strength: reliability and validity. *Commun Methods Meas.* 2011;5(1):48-75.

46. Ipsos. KnowledgePanel Overview. 2018. https://www.ipsos.com/sites/default/files/18-11-53_Overview_v3.pdf, accessed June 2020

47. Couper M. Web surveys: A review of issues and approaches. *Public Opin Q*. 2000;64(4):464-494.

48. Baker LC, Bundorf MK, Singer S, Wagner TH. *Validity of the Survey of Health and Internet and Knowledge Network's Panel and Sampling.* Stanford, CA: Stanford University; 2003.

49. Chang L, Krosnick JA. National surveys via Rdd telephone interviewing versus the Internet: comparing sample representativeness and response quality. *Public Opin Q*. 2009;73(4):641-678.

50. Hauber AB, Johnson FR, Fillit H, et al. Older americans' risk-benefit preferences for modifying the course of alzheimer disease. *Alzheimer Dis Assoc Disord*. 2009;23(1):23-32.

51. Piette JD, Heisler M, Wagner TH. Problems paying out-of-pocket medication costs among older adults with diabetes. *Diabetes Care*. 2004;27(2):384-391.

52. Heiss F, McFadden D, Winter J. Who failed to enroll in medicare part D, and why? early results. *Health Aff (Millwood)*. 2006;25(5):w344-54.

53. Schoenborn NL, Janssen EM, Boyd CM, Bridges JFP, Wolff AC, Pollack CE. Preferred clinician communication about stopping cancer screening among older US adults: results from a national survey. *JAMA Oncol.* 2018;4(8):1126-1128.

54. Lecheler S, de Vreese CH. Getting real: the duration of framing effects. *J Commun.*2011;61(5):959-983.

55. Fishbein M, Ajzen I. Predicting and changing behavior: the reasoned action approach. New York: Psychology Press;2011.

56. Baig S.A., Noar S.M., Gottfredson N.C., Boynton M.H., Ribisl K.M., Brewer N.T. UNC perceived message effectiveness: Validation of a brief scale. *Ann. Behav. Med*. 2019;53(8):732–742.

57. Baig S.A., Noar S.M., Gottfredson N.C., Lazard A.J., Ribisl K.M., Brewer N.T. Incremental criterion validity of message perceptions and effects perceptions in the context of anti-smoking messages. *J. Behav. Med.* 2021;44(1):74–83.

58. Watson D, Clark LA, Tellegen A. Development and validation of brief measures of positive and negative affect: the PANAS scales. *J Pers Soc Psychol.* 1988;54(6):1063-70.

59. Nagler RH, Yzer MC, Rothman AJ. Effects of Media Exposure to Conflicting Information About Mammography: Results From a Population-based Survey Experiment. *Ann Behav Med*. 2019;53(10):896-908.

Office of Human Subjects Research
Institutional Review Boards

1620 McElderry Street, Reed Hall, Suite B-130
Baltimore, Maryland 21205-1911
410-955-3008
410-955-4367 Fax
e-mail: jhmeirb@jhmi.edu

Date: April 19, 2021

APPLICATION ACKNOWLEDGEMENT

**Review Type:**	Exempt
**Principal Investigator:**	Nancy Schoenborn
**Number:**	IRB00283392
**Title:**	Messaging strategies to reduce breast cancer over-screening in older women
**Committee Chair:**	Kenneth Cohen
**IRB Committee:**	IRB-6

Date of acknowledgement: April 19, 2021

Date of expiration: April 18, 2022

The JHM IRB has determined that the above-referenced new application qualifies as exempt research under the DHHS regulations.

If there are changes in this project that may affect this determination, you should consult with the JHM IRB before making those changes.

45 CFR 46.104(d)(2)(ii) - Research that only includes interactions involving educational tests (cognitive, diagnostic, aptitude, achievement), survey procedures, interview procedures, or observation of public behavior (including visual or auditory recording) where any disclosure of the human subjects' responses outside the research would not reasonably place the subjects at risk of criminal or civil liability or be damaging to the subjects' financial standing, employability, educational advancement, or reputation;

This note serves as a reminder that you are obligated to submit a Further Study Action for Changes in Research in order to: 1.) Submit any additional surveys or alterations to the existing survey; and, 2.) Update your funding status to awarded/Certificate of Confidentiality status if and when the award is issued.

To keep the JHM IRB application current we are assigning an Expiration Date as noted above. Prior to the expiration date, you will receive an email notification indicating that some action is required. If the Board has determined that a Continuing Review or Progress Report is required, you will need to submit Continuing Review or Progress Report prior to the expiration date. If the Board has determined that No Progress Report is required, you may run the administrative extend approval function.

IRB review included the following:

Progress Report Required:

The Board determined that this research meets the criteria for submission of a Progress Report as an alternative to a Continuing Review Application. The Progress Report must be submitted using a Further Study Action and selecting progress report at least 6 weeks prior to the expiration date. Please note, the Progress Report **must** be submitted prior to the expiration date shown on this notice. If the Progress Report is not submitted prior to the expiration date all activity must stop. Before any research activity can resume, you must submit the progress report.

Expiration Date: The expiration date for this research is listed above. If a continuing review application is required for this research and the approval lapses, the research must stop and you must submit a request to the IRB to determine whether it is in the best interests of individual participants to continue with protocol-related procedures.

Continuing Review/Progress Report: Continuing Review/Progress Report Applications should be submitted at least 6 weeks prior to the study expiration date.

If a progress report is required, failure to submit a progress report in the time period requested will result in your inability to submit any further study actions other than a progress report until your progress report is submitted and acknowledged.

If a Continuing Review application is required, failure to allow sufficient time for review may result in a lapse of approval. If the Continuing Review Application is not submitted prior to the expiration date, your study will be terminated and a New Application must be submitted to reinitiate the research.

Unanticipated Problems: All unanticipated problems must be submitted using a Protocol Event Report.

If this research has a commercial sponsor, the research may not start until the sponsor and JHU have signed a contract.

The JHMIRB is constituted to meet the requirements of the Privacy Rule at section 45 CFR 164.512(i)(1)(i)(B) and is authorized and qualified to serve as the Privacy Board for human subjects research applications conducted by Hopkins' faculty members. The JHM IRB reviewed your request to waive or alter authorization for the above-referenced project. The IRB determined that all specific criteria for a waiver or alteration of authorization were met, as follows:
(A) The use or disclosure of protected health information involves no more than minimal risk to the privacy of individuals, based on, at least, the presence of the following elements;
(1) An adequate plan to protect the identifiers from improper use and disclosure;
(2) An adequate plan to destroy the identifiers at the earliest opportunity consistent with conduct of the research, unless there is a health or research justification for retaining the identifiers or such retention is otherwise required by law; and
(3) Adequate written assurances that the protected health information will not be reused or disclosed to any other person or entity, except as required by law, for authorized oversight of the research study, or for other research for which the use or disclosure of protected health information would be permitted;
(B) The research could not practicably be conducted without the waiver or alteration; and
(C) the research could not practicably be conducted without access to and use of the protected health information.

Study documents:

Recruitment Materials:
Phone recruitment script for pilot survey.docx

HIPAA Form 4:
form4 messaging .doc

Additional Supplemental Study Documents:
Survey for messaging study.docx

Protocol:

eformE messaging grant.docx

Johns Hopkins Study Team Members:

None

The Johns Hopkins Institutions operate under multiple Federal-Wide Assurances: The Johns Hopkins University School of Medicine - FWA00005752, Johns Hopkins Health System and Johns Hopkins Hospital - FWA00006087

eIRB: FYI - Do not reply - NHSR or Exempt Research, or Research where no Progress Report is Required: Project Extension

nancyli@jhmi.edu <nancyli@jhmi.edu>
Mon 3/21/2022 10:31 AM
To:Nancy Schoenborn <nancyschoenborn@jhmi.edu>

eIRB:<br>FYI - Do not reply	NHSR or Exempt Research, or Research where no Progress Report is Required: Project Extension
**Study Number:**	**IRB00283392**
**Study Name:**	**Messaging strategies to reduce breast cancer over-screening in older women**
**PI:**	**Nancy Schoenborn**
**IRB Committee:**	**IRB-6**
**Link to Workspace:**	**IRB00283392**

The above-referenced eIRB application has been extended for an additional 3 years. The new expiration date is 4/18/2025

QUESTIONS?

Contact:

JHM IRB Office at 410-955-3008
JHM eIRB Help Desk at jhmeirb@jhmi.edu

FYI_Extend Approval
