# Supplementary material for: Effect of Messaging on Support for Breast Cancer Screening Cessation Among Older US Women: A Randomized Clinical Trial
Source: JAMA Netw Open. 2024 Aug 19;7(8):e2428700. doi: 10.1001/jamanetworkopen.2024.28700 (PMC11333986; doi:10.1001/jamanetworkopen.2024.28700)
Supplement: Supplement 2. — eTable 1. Comparison of Responders vs Nonresponders eTable 2. Comparison of Participants Who Completed Both Waves 1 and 2 vs Those Who Completed Wave 1 Only and Did Not Complete Wave 2 eTable 3. Mean Outcome Scores Regarding Stopping Screening Intention for Oneself Among Those With Higher Breast Cancer Risk eAppendix. Survey Instrument [file jamanetwopen-e2428700-s002.pdf]

Supplementary Online Content

Schoenborn NL, Gollust SE, Nagler RH, et al. Effect of messaging on support for breast cancer screening cessation among older us women: a randomized clinical trial. *JAMA Netw Open*. 2024;7(8):e2428700. doi:10.1001/jamanetworkopen.2024.28700

eTable 1. Comparison of Responders vs Nonresponders

eTable 2. Comparison of Participants Who Completed Both Waves 1 and 2 vs Those Who Completed Wave 1 Only and Did Not Complete Wave 2

eTable 3. Mean Outcome Scores Regarding Stopping Screening Intention for Oneself Among Those With Higher Breast Cancer Risk

eAppendix. Survey Instrument

This supplementary material has been provided by the authors to give readers additional information about their work.

© 2024 Schoenborn NL et al. *JAMA Network Open*.

eTable 1. Comparison of Responders vs Nonresponders

	Responders, No. (%), n=5108	Non-responders No. (%), n=1954	P-value
Age, mean (SD)	72.7 (5.9)	72.4 (6.3)	0.04
Race			
Black, non-Hispanic	436 (8.5)	227 (11.6)	<0.001
Hispanic	247 (4.8)	145 (7.4)	
White, non-Hispanic	4221 (82.6)	1500 (76.8)	
Other [a]	204 (4.0)	82 (4.2)	
Education			
High school or less	1435 (28.1)	526 (26.9)	0.32
College or more	3673 (71.9)	1428 (73.1)	
Geographic region			
Northeast	884 (17.3)	360 (18.4)	0.36
Midwest	1219 (23.9)	473 (24.2)	
South	1832 (35.9)	708 (36.2)	
West	1173 (23.0)	413 (21.1)	

[a] "Other" included non-Hispanic 2+ races and non-Hispanic other race.

© 2024 Schoenborn NL et al. *JAMA Network Open*.

eTable 2. Comparison of Participants Who Completed Both Waves 1 and 2 vs Those Who Completed Wave 1 Only and Did Not Complete Wave 2

Participant characteristics	Completed waves 1 and 2, No. (%), n=2796	Completed wave 1 but not wave 2, No. (%), n=255	P-value
Age			0.97
65 to <75	1850 (66.2)	169 (66.3)	
75+	946 (33.8)	86 (33.7)	
Life expectancy [a]			0.68
10+ years	2235 (81.5)	202 (80.5)	
<10 years	506 (18.5)	49 (19.5)	
Race			0.22
Black, non-Hispanic	246 (8.8)	26 (10.2)	
Hispanic	131 (4.7)	17 (6.7)	
White, non-Hispanic	2308 (82.6)	198 (77.7)	
Other [b]	111 (4.0)	14 (5.5)	
Geographic region			0.29
Northeast	473 (16.9)	52 (20.4)	
Midwest	668 (23.9)	55 (21.6)	
South	1004 (35.9)	82 (32.2)	
West	651 (23.3)	66 (25.9)	
Ever had mammogram	2707 (96.8)	251 (98.4)	0.15
Self-reported mammogram within last 2 years	2223 (79.6)	212 (83.1)	0.18
Cancer worry			0.002
Somewhat, a little or not at all worried	2553 (91.5)	219 (85.9)	
Moderately or extremely worried	236 (8.5)	36 (14.1)	
Family history of breast cancer			0.08
None	2085 (76.6)	175 (70.6)	
One	518 (19.1)	58 (23.4)	
Two or more	115 (4.2)	15 (6.1)	
Education			0.12
Less than high school	64 (2.3)	7 (2.8)	
High school	738 (26.4)	51 (20.0)	
Some college	950 (34.0)	100 (39.2)	
Bachelor's degree or more	1044 (37.3)	97 (38.0)	
Low health literacy [c]	219 (7.9)	20 (7.9)	1.00
Breast cancer risk, mean (SD) [27]	2.4 (1.3)	2.4 (1.3)	0.67

[a] Life expectancy was estimated using the Schonberg mortality index.[25] Scores for participants ranged from 0 to 19. Scores ≥10 are associated with >50% chance of 10-year mortality. Thus, women who score ≥10 are estimated to have <10- year life expectancy.

[b] "Other" included non-Hispanic 2+ races and non-Hispanic other race.

[c] Health literacy was assessed in a single validated question – "How confident are you filling out medical forms?"[30] Responses of "not at all", a little bit", "somewhat" confident were categorized as low health literacy; responses of "quite a bit" and "extremely" confident were categorized as normal health literacy.

© 2024 Schoenborn NL et al. *JAMA Network Open*.

eTable 3. Mean Outcome Scores Regarding Stopping Screening Intention for Oneself Among Those With Higher Breast Cancer Risk[a,b]

		Group 1: No message (wave 1 n=162; wave 2 n=143)	Group 2: message from clinician at wave 1, no message at wave 2 (wave 1 n=163; wave 2 n=151)	Group 3: messages from news story at wave 1 and clinician at wave 2 (wave 1 n=149; wave 2 n=134)	Group 4: messages from family member at wave 1 and clinician at wave 2 (wave 1 n=165; wave 2 n=153)
Stopping screening intention for oneself, 5-year breast cancer risk $\geq$ 3%	Wave 1	2.32	2.18	2.06	2.21
	# Missing.	1	0	0	0
	Wave 2	1.97	2.01	2.10	2.96*†
	# Missing	0	0	1	1

[a] Both outcomes were measured on a 7-point scale. Higher score indicates higher intention to stop screening for oneself (1=very likely to get a mammogram, 7=very unlikely to get a mammogram).

[b] Comparisons among the experimental groups at wave 2 used ANOVA with Tukey test and are denoted as follows: * denotes that the mean was significantly different when compared with Group 1 that received no messages (p≤0.05); † denotes that the mean was significantly different than Group 2 that received a single message from clinician (p≤0.05). There was no significant difference between Groups 3 and 4 for any of the comparisons.

© 2024 Schoenborn NL et al. *JAMA Network Open*.

eAppendix. Survey Instrument

Wave 1

Sharing information so women 65 years and older can make informed decisions about breast cancer screening

The goal of this survey is to understand how to communicate information about breast cancer screening so women 65 years and older can make decisions that are right for them. In this survey, we want to learn from you about the best ways to share what is known about breast cancer screening.

This is the first of a two-part survey. This survey takes about 15 minutes to complete. There are no right or wrong answers. We want your honest opinions and feelings. Your completion of this survey will serve as your consent to be in this research study. Your answers will not be traced back to you. In 2 weeks, we will send you a shorter follow-up survey.

Q1. Have you ever been told by a doctor that you have breast cancer?

1. Yes
2. No

(Not qualify for the study if response =1 or refused)

Now we are going to ask you some questions about your own personal history of getting checked for breast cancer.

Breast cancer screening means checking a woman's breasts for cancer when she is not having any breast problems. A common breast cancer screening test is the mammogram, which is an x-ray picture of the breast.

Q2. Have you ever had a mammogram?

1. Yes
2. No

Q3. When was your last mammogram?
1. A year ago or less
2. More than 1 year ago, up to 2 years ago
3. More than 2 years ago, up to 5 years ago
4. More than 5 years ago

Q4. Have you ever had an abnormal mammogram? An abnormal mammogram would require that you go back for more mammograms or other testing.
1. Yes
2. No

Q5. Have you ever had a breast biopsy?

© 2024 Schoenborn NL et al. *JAMA Network Open*.

1. Yes
2. No

Q6. How many breast biopsies have you had?

1. 1
2. 2 or more

We want to know your opinion about mammograms. We are referring to mammograms that are done for screening – to look for cancer when a woman is not having any breast problems. We are NOT talking about mammograms that are done to look into a problem, such as breast pain.

We will now tell you about an example person, Ms. Johnson.

Ms. Johnson is 75 years old. She has multiple serious health problems including heart disease, kidney disease, diabetes, and arthritis. She takes several medications and has been in the hospital twice in the last year. Because of her health, she can no longer shop by herself or drive. She walks with a cane. She enjoys reading the news, connecting with friends on Facebook, playing cards, and spending time with her family. Ms. Johnson has had regular mammograms. She has no history of breast cancer and her last mammogram two years ago was normal.

[**if Group 2**]
Ms. Johnson regularly sees her primary care doctor, Dr. Smith. During a recent routine follow-up visit, Dr. Smith shared some information about mammograms.

Below is the information that Dr. Smith shared with Ms. Johnson about mammograms. Please read what Dr. Smith shared and answer the questions that follow.

> *"Medical guidelines recommend against regular mammograms for women over age 65 who have a lot of health problems because there are more downsides than benefits. I have several older patients who have had a false alarm from their mammograms – their mammograms showed abnormal results but really there was no cancer. These women were very distressed by the abnormal results while waiting to find out if they had cancer. They also had to get breast biopsies that were painful.*
>
> *Even if cancer was found, many breast cancers found by mammograms are so slow growing that they never would have caused problems. In these cases, women end up getting cancer treatments they would not have needed and can have side effects from the surgery, radiation, or medication treatments.*
>
> *For some women over age 65 who have a lot of health problems, mammograms could have more downsides than benefits. It may be worth considering stopping mammograms."*

[**if Group 3**]
Ms. Johnson reads USA Today news stories regularly on the computer. She recently learned some information from a news story about mammograms for older women.

© 2024 Schoenborn NL et al. *JAMA Network Open*.

Below is the information that Ms. Johnson learned from the USA Today news story about mammograms. Please read this information and answer the questions that follow.

> *"Medical guidelines recommend against regular mammograms for women over age 65 who have a lot of health problems because there are more downsides than benefits. The journalist interviewed several older women who recently had a false alarm from their mammograms – their mammograms showed abnormal results but really there was no cancer. These women were very distressed by the abnormal results while waiting to find out if they had cancer. They also had to get breast biopsies that were painful.*
>
> *Even if cancer was found, many breast cancers found by mammograms are so slow growing that they never would have caused problems. In these cases, women end up getting cancer treatments they would not have needed and can have side effects from the surgery, radiation, or medication treatments.*
>
> *For some women over age 65 who have a lot of health problems, mammograms could have more downsides than benefits. It may be worth considering stopping mammograms."*

[**if Group 4**]
Ms. Johnson has a close family member, Linda, who comes and visits her on weekends. During a recent visit, Ms. Johnson mentioned to Linda that she had a doctor's appointment for a check-up in the next week. During this conversation with Linda, the topic of mammograms came up. Linda shared some information she had recently heard about mammograms.

Below is the information that Linda shared with Ms. Johnson about mammograms. Please read what Linda shared and answer the questions that follow.

> *"I read that medical guidelines recommend against regular mammograms for women over age 65 who have a lot of health problems because there are more downsides than benefits. I know several older women who recently had a false alarm from their mammograms – their mammograms showed abnormal results but really there was no cancer. These women were very distressed by the abnormal results while waiting to find out if they had cancer. They also had to get breast biopsies that were painful.*
>
> *I heard that even if cancer was found, many breast cancers found by mammograms are so slow growing that they never would have caused problems. In these cases, women end up getting cancer treatments they would not have needed and can have side effects from the surgery, radiation, or medication treatments.*
>
> *For some women over age 65 who have a lot of health problems, mammograms could have more downsides than benefits. It may be worth considering stopping mammograms."*

© 2024 Schoenborn NL et al. *JAMA Network Open*.

Q7. How strongly do you believe that older women with a lot of health problems like Ms. Johnson should get a mammogram in the next two years?

1 Definitely should get a mammogram
2
3
4
5
6
7 Definitely should not get a mammogram

Q8. How likely is it that you will get a mammogram in the next two years?

1 Very likely
2
3
4
5
6
7 Very unlikely

Q9. The next question includes words that describe different feelings and emotions. Please indicate how you feel after reading the information that was shared.

Annoyed

1 Not at all
2
3
4
5 Extremely

Worried

1 Not at all
2
3
4
5 Extremely

Q10. Thinking about the information that was shared, please rate how much you agree or disagree with the following statements.

1. This information discourages me from wanting to get a mammogram.
2. This information makes getting a mammogram seem unpleasant to me.
3. This information makes me concerned about the health effects of getting a mammogram.

© 2024 Schoenborn NL et al. *JAMA Network Open*.

Answers in column:

1. Strongly disagree
2. Somewhat disagree
3. Neither agree nor disagree
4. Somewhat agree
5. Strongly agree

A woman’s family history and experience with menstrual periods and childbirth are some of the factors that can affect her risk of developing breast cancer. The next few questions help us better understand your risk.

Q11. What was your age when you had your first menstrual period?

1. 7-11
2. 12-13
3. 14+
4. Don’t know

Q12. What was your age when you gave birth to your first child?

1. No births
2. <20
3. 20-24
4. 25-29
5. 30+
6. Don’t know

Q13. How many of your first-degree relatives (mother, sisters, daughters) have had breast cancer?

1. None
2. One
3. More than one
4. Don’t know

Q14. How worried are you about getting breast cancer?

1. Not at all
2. Slightly
3. Somewhat
4. Moderately
5. Extremely

Lastly, we are going to ask some questions about your health and your thoughts about healthcare.

Q15. How confident are you in filling out medical forms by yourself?

© 2024 Schoenborn NL et al. *JAMA Network Open*.

1. Extremely
2. Quite a bit
3. Somewhat
4. A little bit
5. Not at all

Q16. Has a doctor ever told you that you have any of the following health conditions?

Statements in row:
1. Diabetes or high blood sugar
2. Cancer of any kind (except minor skin cancers)?
3. Chronic lung disease (COPD), such as emphysema or chronic bronchitis?

Answers in column:
1. Yes
2. No

Q17. Do you have difficulty walking ¼ mile (several city blocks)?

1. Yes
2. No

Q18. Do you need the help of others in everyday household chores, doing necessary business, shopping, or getting around, because of a physical, mental or emotional problem?

1. Yes
2. No

Q19. During the past 12 months, how many times have you been hospitalized overnight?

1. None
2. Once
3. Twice or more

© 2024 Schoenborn NL et al. *JAMA Network Open*.

Wave 2

Sharing information so women 65 years and older can make informed decisions about breast cancer screening

The goal of this survey is to understand how to communicate information about breast cancer screening so women 65 years and older can make decisions that are right for them. In this survey, we want to learn from you about the best ways to share what is known about breast cancer screening.

This is the second of a two-part survey. Thank you for responding to the first survey about 2 weeks ago. We have some additional questions. Please note that while some of the information and questions may be similar to what you saw in the earlier survey, it is important to get your responses to these questions again.

Today's survey takes about 10 minutes to complete. There are no right or wrong answers. We want your honest opinions and feelings. Your completion of this survey will serve as your consent to be in this research study. Your answers will not be traced back to you.

We want to know your opinion about mammograms. We are referring to mammograms that are done for screening – to look for cancer when a woman is not having any breast problems. We are NOT talking about mammograms that are done to look into a problem, such as breast pain.

In an earlier survey you completed 2 weeks ago, we told you about an example person, Ms. Johnson. To refresh your memory, below is the same description of Ms. Johnson we showed you before.

Ms. Johnson is 75 years old. She has multiple serious health problems including heart disease, kidney disease, diabetes, and arthritis. She takes several medications and has been in the hospital twice in the last year. Because of her health, she can no longer shop by herself or drive. She walks with a cane. She enjoys reading the news, connecting with friends on Facebook, playing cards, and spending time with her family. Ms. Johnson has had regular mammograms. She has no history of breast cancer and her last mammogram two years ago was normal.

[If Group 3 or Group 4]
Now Ms. Johnson is at a routine follow up visit with her primary care doctor, Dr. Smith, and Dr. Smith shared some information about mammograms.

Below is the information that Dr. Smith shared with Ms. Johnson about mammograms. Please read the information and answer the questions that follow.

> *"Medical guidelines recommend against regular mammograms for women over age 65 who have a lot of health problems because there are more downsides than benefits. I have several older patients who have had a false alarm from their mammograms – their mammograms showed abnormal results but really there was no cancer. These women were very distressed by the abnormal results while waiting to find out if they had cancer. They also had to get breast biopsies that were painful.*

© 2024 Schoenborn NL et al. *JAMA Network Open*.

Even if cancer was found, many breast cancers found by mammograms are so slow growing that they never would have caused problems. In these cases, women end up getting cancer treatments they would not have needed and can have side effects from the surgery, radiation, or medication treatments.

For some women over age 65 who have a lot of health problems, mammograms could have more downsides than benefits. It may be worth considering stopping mammograms."

How strongly do you feel that older women with a lot of health problems like Ms. Johnson should get a mammogram in the next two years?

1 Definitely should get a mammogram
2
3
4
5
6
7 Definitely should not get a mammogram

How likely is it that you will get a mammogram in the next two years?

1 Very unlikely
2
3
4
5
6
7 Very likely

If you would like to learn more about this topic, tools are available for helping women ages 75 or older to make decisions about mammograms. There are currently no available tools tailored for women ages 65-74.

- Ages 75-84 mammogram decision aid: https://eprognosis.ucsf.edu/decision_aids/Mammography_75-84.pdf

- Ages 85 or older mammogram decision aid: https://eprognosis.ucsf.edu/decision_aids/Mammography_85.pdf

© 2024 Schoenborn NL et al. *JAMA Network Open*.
