# Supplementary material for: Effect of Messaging on Support for Breast Cancer Screening Cessation Among Older US Women: A Randomized Clinical Trial
Source: JAMA Netw Open. 2024 Aug 19;7(8):e2428700. doi: 10.1001/jamanetworkopen.2024.28700 (PMC11333986; doi:10.1001/jamanetworkopen.2024.28700)
Supplement: Supplement 3. — Data Sharing Statement [file jamanetwopen-e2428700-s003.pdf]

Data Sharing Statement

Schoenborn. Effect of Messaging on Support for Breast Cancer Screening Cessation Among Older US Women. *JAMA Netw Open*. Published August 19, 2024.
doi:10.1001/jamanetworkopen.2024.28700

Data
Data available: Yes
Data types: Deidentified participant data, Data dictionary
How to access data: The datasets generated and/or analyzed during the current study are not publicly available to maintain participant confidentiality but are available from the corresponding author on reasonable request.
When available: With publication

Supporting Documents
Document types: None

Additional Information
Who can access the data: Researchers whose proposed use of the data has been approved.
Types of analyses: Secondary analyses
Mechanisms of data availability: without investigator support after approval of a proposal with a signed data access agreement.
